# The Prediction of the In Vitro Release Curves for PLGA-Based Drug Delivery Systems with Neural Networks

**DOI:** 10.3390/pharmaceutics17040513

**Published:** 2025-04-14

**Authors:** Zheng Zhang, Bolun Zhang, Ren Chen, Qian Zhang, Kangjun Wang

**Affiliations:** 1 College of Chemical Engineering, Shenyang University of Chemical Technology, Shenyang 110142, China; zhengzhang@syuct.edu.cn (Z.Z.); dandanbaixue@outlook.com (B.Z.); 2 School of Software, Dalian University of Technology, Dalian 116620, China; g2706@mail.dlut.edu.cn

**Keywords:** PLGA carriers, neural networks, data-driven modeling, drug release prediction, in vitro release curves

## Abstract

**Background/Objectives:** The accurate prediction of drug release profiles from Poly (lactic-co-glycolic acid) (PLGA)-based drug delivery systems is a critical challenge in pharmaceutical research. Traditional methods, such as the Korsmeyer-Peppas and Weibull models, have been widely used to describe in vitro drug release kinetics. However, these models are limited by their reliance on fixed mathematical forms, which may not capture the complex and nonlinear nature of drug release behavior in diverse PLGA-based systems. **Method:** In response to these limitations, we propose a novel approach—DrugNet, a data-driven model based on a multilayer perceptron (MLP) neural network, aiming to predict the drug release data at unknown time points by fitting release curves using the key physicochemical characteristics of PLGA carriers and drug molecules, as well as in vitro drug release data. We establish a dataset through a literature review, and the model is trained and validated to determine its effectiveness in predicting different drug release curves. **Results:** Compared to the traditional Korsmeyer–Peppas and Weibull semi-empirical models, the MSE of DrugNet decreases by 20.994 and 1.561, respectively, and ($R^{2}$) increases by 0.036 and 0.005. **Conclusions:** These results demonstrate that DrugNet has a stronger ability to fit drug release curves and better capture nonlinear relationships in drug release data. It can deal with the nonlinear change of data better, has stronger adaptability and advantages than traditional models, and overcomes the limitations of the mathematical expressions in traditional models.

## 1. Introduction

Drug delivery carriers (including liposomes, polymers, inorganic nanomaterials, and other types) promote the development of precision medicine by optimizing the efficiency and targeting of drug delivery. Among these, Poly(lactic-co-glycolic acid) (PLGA), as a drug excipient approved by the Food and Drug Administration (FDA), is one of the most widely used biodegradable polymers in drug delivery systems due to its versatile properties, such as its biocompatibility, biodegradability, and controlled release characteristics [1,2,3,4]. These PLGA systems are of great significance for the sustained release of drugs, as they can degrade over time and release encapsulated drugs in a controlled manner. As such, accurately predicting the drug release behavior in PLGA-based systems is critical for the design of effective drug delivery strategies, reducing the costs of trial and error, ensuring the optimal therapeutic effect, and minimizing potential side effects [5].

The traditional methods for simulating the drug release in PLGA systems typically involve semi-empirical models such as the Korsmeyer–Peppas (K-P) and Weibull models. These models fit the curve for the drug release in vitro by fitting experimental data to specific mathematical pharmacokinetic formulas [6]. The K-P model characterizes the release curve through a power exponential equation, while the Weibull model is favored for its flexibility in representing both Fick and non-Fick diffusion processes [7]. However, these empirical models are limited by their mathematical forms and number of parameters, which affect their applicability under different drug carrier combinations.

The limitations of traditional semi-empirical models have sparked interest in machine learning (ML) techniques as potential solutions. In particular, neural networks have gained attention for their ability to model complex nonlinear relationships, providing an effective approach to addressing the challenges in drug delivery systems [8,9,10]. Rafienia et al. [11] were pioneers in using feedforward neural networks to predict the release of corticosteroid drugs, demonstrating that an MLP has higher reliability and efficiency compared to those of other models. Petrović et al. [12] employed Elman dynamic neural networks to model the dissolution profiles of hydrophilic and lipid matrix tablets, successfully surpassing static networks like MLPs and further promoting the development of neural networks in the field of drug release. More recently, Koshari et al. [13] proposed a data-driven approach combining stochastic optimization algorithms to develop predictive models for sustained drug release, successfully designing zero-order release profiles. These studies highlight the continuous progress of neural networks in enhancing the accuracy and control of drug release predictions.

**Figure 1:**
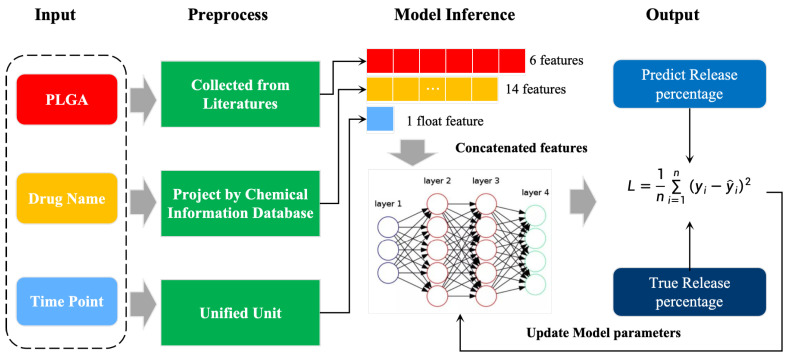
Illustration of the overall scheme process.

However, these applications have neglected to pay attention to the effects of the carrier and the drug itself and their interactions on the drug release, so we built a model for in vitro drug release curves, DrugNet, to fit these different drug release curves in vitro based on the PLGA carriers (Figure 1). This research aims to explore the potential of a neural network model for fitting the in vitro drug release profiles of PLGA-based systems. Specifically, we collected six PLGA characteristics that affect the drug release in vitro through research in the literature, including molecular weight, PDI, particle size, zeta potential, monomer ratio, and encapsulation efficiency. Moreover, we combined the collected dataset with the drug names to create a dataset dedicated to fitting the curves for in vitro drug release, which can provide data support for subsequent work. In addition, DrugNet extracted key information features on the drugs from a chemical information database and combined the data features for PGLA and predicted time points as the input to the model for curve fitting training. With the neural network model, we avoid formalized prediction results and can fit multiple types of drug release curves.

## 2. Materials and Methods

The characteristics of the PLGA carriers and the drug molecular descriptors used in this study are obtained from the literature and chemical information libraries. We select several key features of PLGA carriers as inputs, including their molecular weight, polydispersity index (PDI), particle size, zeta potential, monomer ratio, and encapsulation efficiency. These features affect the drug release behavior of PLGA carriers, and therefore, they are also important features in our model training process.

### 2.1. PLGA Carrier Characteristics

We collect 39 sets of PLGA carrier characterization data from the existing literature, and each set of data contains the six characteristics mentioned above, each of which plays an important role. Among these, the molecular weight and PDI of PLGA affect the rate and control of drug release, while the particle size and zeta potential are related to the stability of the carrier and the uniformity of drug release. Additionally, the monomer ratio and the encapsulation efficiency also affect the drug loading and the controlled release performance of the system. In this study, all of the above features are used as inputs to our neural network model.

### 2.2. Drug Molecular Descriptors

Our drug molecular descriptors are obtained in a two-step process by first retrieving drug information from the PubChem database using the drugs’ names, followed by calculating the corresponding molecular descriptors using the RDKit toolset. The descriptors used in this study include molecular weight, LogP (hydrophobicity), hydrogen bond donors, hydrogen bond acceptors, heavy atom count, rotatable bonds, polar surface area (TPSA), and Lipinski’s Rule of Five (e.g., molecular weight ≤ 500, LogP ≤ 5). These descriptors provide structural information on and the physicochemical characteristics of drug molecules that are relevant to the drug release behavior and likewise serve as inputs to our model.

### 2.3. The Dataset Construction and Experimental Conditions

In addition to the carrier and drug features mentioned above, we manually obtain 39 sets of in vitro drug release sample data from the literature and construct datasets for model training. Each sample in the dataset consists of six-dimensional carrier features, fourteen-dimensional drug features, and multiple time points with corresponding release percentages. The significant differences between the time scales and the number of time points of different samples in the dataset pose a great challenge to the generalization ability of our model. Regarding the experimental conditions, most of the data are obtained under typical in vitro conditions (using Phosphate-Buffered Saline solution at a pH of 7.4 and 37 °C) which are commonly used to simulate drug release environments. The details on the dataset are shown in Table 1.

### 2.4. Model Design and Training

In this study, a multilayer perceptron (MLP) neural network model is used to fit the in vitro drug release curves based on the PLGA carrier features, the drug characteristics, and a subset of in vitro release data points. The input features of the model include 6 physicochemical characteristics of the PLGA carrier (i.e., molecular weight, PDI, particle size, zeta potential, monomer ratio, encapsulation efficiency), 14 characteristics of the drug (e.g., molecular weight, LogP, hydrogen bond donors/acceptors, etc.), and a single time point for the drug release. Our selected carrier characteristics and drug characteristics have potential impacts on the drug release behavior, which is crucial for our drug release predictions. Our code is available at https://github.com/g2706/plga, accessed on 6 April 2025.

In the data preprocessing stage, we use an average estimation strategy for each feature to deal with missing values, followed by normalization of the dataset. As for the partitioning of the datasets, we employ a time-point-based overall data splitting approach to model training rather than the traditional sample-based data splitting method. Specifically, we combine the features of all of the samples with the corresponding time point data to form a unified input dataset, which is then used for training. During the training process, 80% of the data is used for training, while the remaining 20% is used as a test set for model validation. However, a key characteristic of this data splitting approach is that the time point data for all samples are involved in the training process. This means that although not all time points for each individual sample are included in training, every sample’s data in the drug release curve have "seen" data from other samples during training.

The neural network architecture employs a standard MLP structure. Specifically, the input layer consists of nodes corresponding to the number of input features, while the number of hidden nodes is determined through appropriate tuning to achieve a good fitting performance. The output layer contains a single node representing the predicted drug release percentage. The model is trained using the mean squared error (MSE) as the loss function. The model’s fitting performance was evaluated using both the MSE and the coefficient of determination ($R^{2}$). The MSE is a common evaluation metric for regression models used to measure the difference between the predicted and actual values. A smaller MSE indicates the better performance of the model. The formula for the MSE is Equation (1):

(1) $$MSE=\frac{1}{n}\sum_{i=1}^{n}{\left(y_{i}-\hat{y_{i}}\right)}^{2}$$

where *n* is the number of samples, $y_{i}$ is the actual value, and $\hat{y_{i}}$ is the predicted value. The MSE reflects the deviation in the model’s predictions, providing an intuitive measure of the prediction error. For example, the drug release is measured in the range of 0–100%, so an MSE of 20 means that the square of the difference between the release value predicted by the model and the actual release value at an average time point is 20. $R^{2}$ is an important metric for evaluating the goodness of fit of a regression model. It measures the proportion of the variance in the dependent variable explained by the model. $R^{2}$ values range from 0 to 1, with a higher value indicating the better performance of the model. The formula for $R^{2}$ is Equation (2):

(2) $$R^{2}=1-\frac{\sum_{i=1}^{n}{\left(y_{i}-\hat{y_{i}}\right)}^{2}}{\sum_{i=1}^{n}{\left(y_{i}-\bar{y}\right)}^{2}}$$

where $y_{i}$ is the actual value, $\hat{y_{i}}$ is the predicted value, and $\bar{y}$ is the mean of the actual values. The closer the $R^{2}$ value is to 1, the better the model explains the variance in the data.

### 2.5. Comparative Semi-Empirical Models

To evaluate the performance of the proposed neural network model, we introduced two traditional semi-empirical models, Korsmeyer–Peppas and Weibull models, as comparisons. These models are widely used to describe drug release kinetics and have good mathematical expressiveness. They are also capable of accurately fitting the release patterns of different drugs. The Korsmeyer–Peppas model [7] is widely used to characterize drug release kinetics, particularly for drugs with irregular or nonlinear release patterns. The basic equation for the Korsmeyer–Peppas model is Equation (3):

(3) $$\frac{M_{t}}{M_{\infty }}=kt^{n}$$

where $M_{t}$ is the amount of drug released at time *t*, $M_{\infty }$ is the total amount of the drug, *t* is time, *k* is the release rate constant, and *n* is the release exponent. By fitting the experimental data, the Korsmeyer–Peppas model estimates the values of *k* and *n*. The release exponent *n* helps to determine the release mechanism. Specifically, if n≤0.5, the release is governed by Fickian diffusion; if 0.5<n<1, the release is controlled by both diffusion and polymer swelling; and if n=1, the release is mainly controlled by Case II diffusion. The Weibull model [7] is a widely used semi-empirical model for describing drug release kinetics. The basic equation for the Weibull model is Equation (4):

(4) $$\frac{M_{t}}{M_{\infty }}=1-\exp\left(-{\left(\frac{t}{\tau }\right)}^{\beta }\right)$$

where β is the shape parameter, τ is the scale parameter, *t* is time, $M_{t}$ is the amount of the drug released at time *t*, and $M_{\infty }$ is the total amount of the drug. The advantage of the Weibull model is its flexibility, allowing it to adapt to different release patterns. When β=1, the model represents a first-order release process; when β<1, it represents diffusion-controlled release; and when β>1, this suggests that the release is influenced by other factors, such as swelling or degradation. The Korsmeyer–Peppas and Weibull models were fitted to the 39 sets of in vitro drug release data, and the corresponding parameters were estimated in order to compare the curve fitting performance with that of the neural network model.

## 3. Results and Discussion

In this study, we compared the performance of the proposed neural network model with that of the traditional Korsmeyer–Peppas and Weibull semi-empirical models in fitting PLGA vector drug release curves in vitro, mainly using the MSE and $R^{2}$ to evaluate the model.

### 3.1. Training and Verification Results for Neural Networks

**Figure 2:**
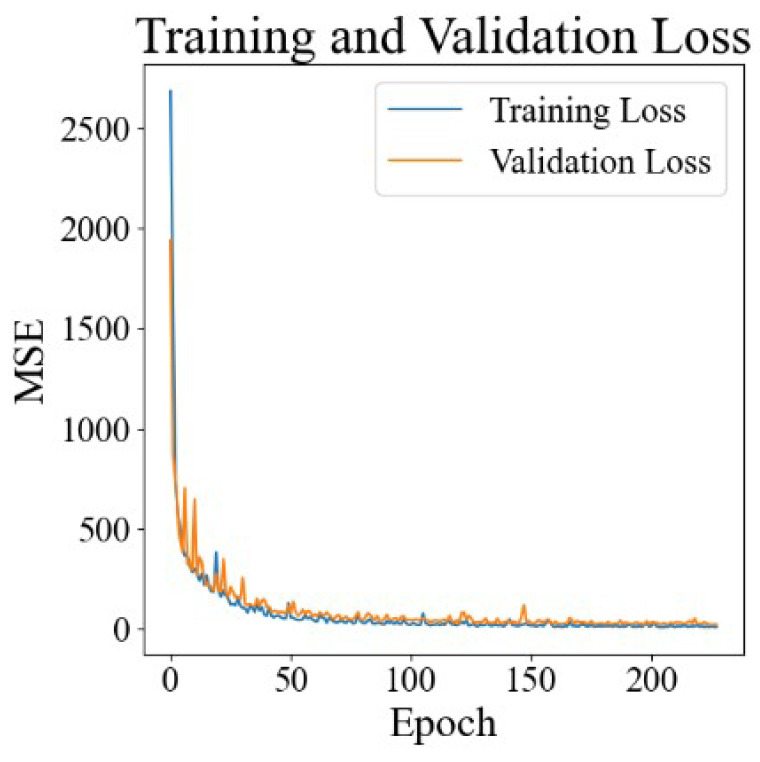
The results of DrugNet loss.

We used 6-dimensional PLGA carrier feature data and 14-dimensional drug molecule feature data obtained in studies from the literature and a time point as the model inputs, divided the dataset according to the time point (see Section 2.4), trained and verified the model, and obtained a loss function diagram, as shown in Figure 2. It is obvious that the training loss and validation loss converge rapidly from about 2000 to about 20, and there is no significant gap between them, indicating that the model can quickly learn effective features and rules from the training data. The rapid decline in and stabilization of the validation losses indicate that the model not only performs well on training data but also maintains a good performance on previously unseen validation data.

### 3.2. Comparison Between DrugNet and Semi-Empirical Models

#### 3.2.1. The Semi-Empirical Models

Pourtaebi Jahromi et al. [7] comprehensively compared various kinetic models for the drug release from PLGA-based nanoparticles, demonstrating that the Weibull model fit the release data best due to its flexibility and high accuracy. Therefore, the Weibull and Korsmeyer–Peppas models were used to fit each sample in the dataset, and the corresponding MSE and $R^{2}$ values were calculated; the results are given in Table 2 and Table 3. The Korsmeyer–Peppas model has an average MSE of 34.327 and an average $R^{2}$ of 0.934. The average MSE of the Weibull model is 14.894, and the average $R^{2}$ is 0.965. The MSE of the Weibull model is 19.422 lower than that of the Korsmeyer–Peppas model, and the $R^{2}$ value is 0.031 higher, which proves that the error between the predicted value and the actual value is smaller, the degree of fitting is higher, and the flexibility is higher.

#### 3.2.2. DrugNet

Next, we trained the neural network model to compute the average MSE and $R^{2}$ values. The mean MSE of the neural network model is 13.333, and the mean $R^{2}$ is 0.970 (Table 2 and Table 3). From these two evaluation indicators, compared with the Korsmeyer–Peppas and Weibull models, the MSE of the neural network model decreases by 20.994 and 1.561, respectively, indicating a smaller gap between the predicted value and the actual value for the neural network model. $R^{2}$ increases by 0.036 and 0.005, respectively, indicating that the model can explain the changes in the dependent variables better and fit the drug release curve better.

**Figure 3:**
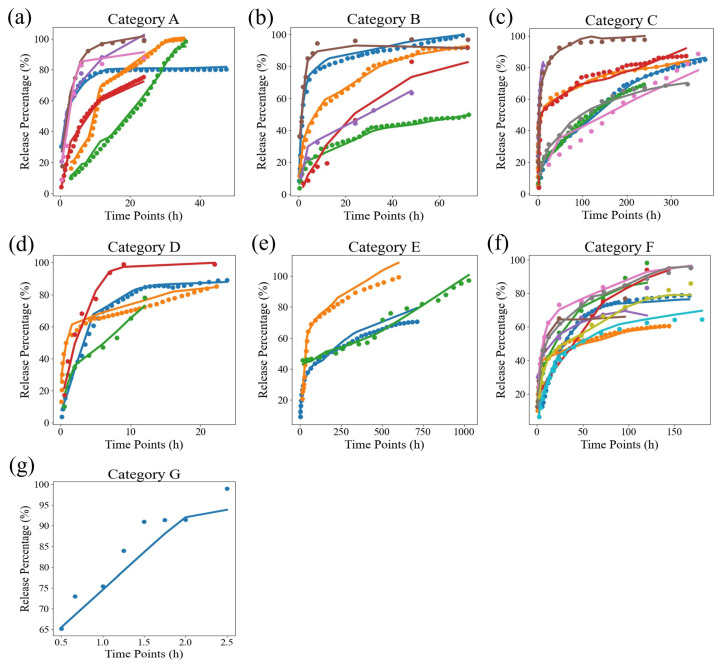
The results of DrugNet. The names A, B, …, and G represent drugs belonging to different release time scales. Specifically, (**a**) is release time in 20–40 (h), (**b**) is release time in 40–100 (h), (**c**) is release time in 200–400 (h), (**d**) is release time in 10–20 (h), (**e**) is release time in 400–1000 (h), (**f**) is release time in 100–200 (h), and (**g**) is release time in 0–10 (h). The discrete points are the true values, and the curves are the predicted values.

Meanwhile, we also calculated the standard deviations in the MSE and $R^{2}$ values for the three models on all samples, as shown in Table 4. The standard deviations in the MSE and $R^{2}$ were 12.662 and 0.031, respectively, which were 3.22 and 0.015 lower than these values for the best semi-empirical model, the Weibull model. This indicates that our model’s results have less variability than that in the Korsmeyer–Peppas and Weibull models, demonstrating the stability of neural networks in fitting drug release curves for different samples. Due to the significant differences in the time scales of the samples, in order to demonstrate the fitting effect of the neural network more clearly, we simply divided the samples based on their release time scales and visualized them, as shown in Figure 3. The discrete points are the actual data, while the curves are the predicted values, which can be divided into seven release time scale ranges: A, B, C, D, E, F, and G. Among these, the time scale of G is the smallest, at about 0–2.5 h, and the time scale of E is the largest, at about 0–1000 h. From the graph, it can be seen that although the time span is large, the overall fitting effect of DrugNet is excellent.

**Figure 4:**
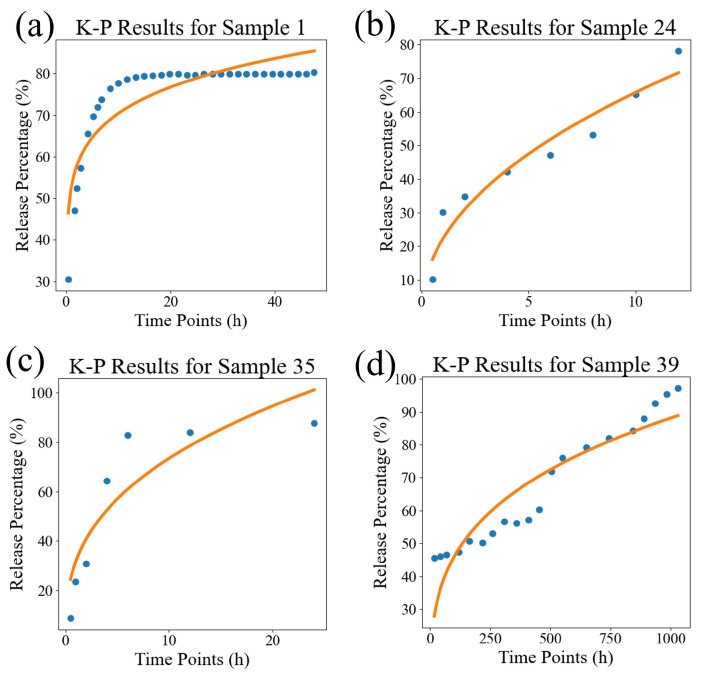
The results of the K-P model. (**a**) The result for sample 1, (**b**) the result for sample 24, (**c**) the result for sample 35, and (**d**) the result for sample 39. The discrete points are the true values, and the curves are the predicted values.

**Figure 5:**
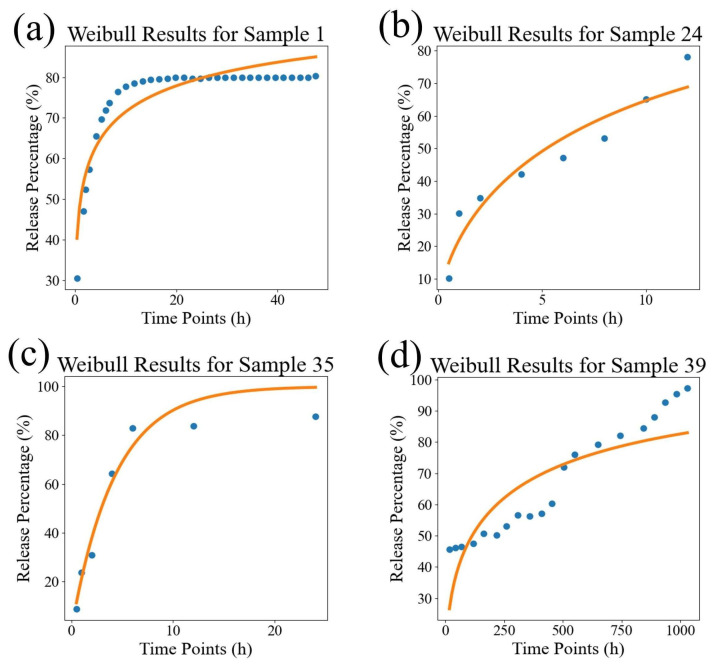
The results of the Weibull model. (**a**) The result for sample 1, (**b**) the result for sample 24, (**c**) the result for sample 35, and (**d**) the result for sample 39. The discrete points are the true values, and the curves are the predicted values.

**Figure 6:**
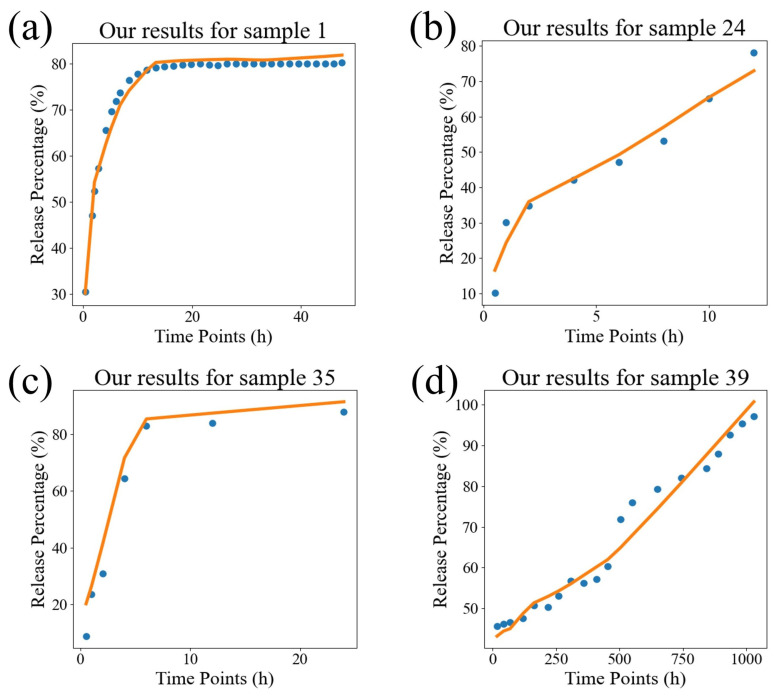
The results of the DrugNet model. (**a**) The result for sample 1, (**b**) the result for sample 24, (**c**) the result for sample 35, and (**d**) the result for sample 39. The discrete points are the true values, and the curves are the predicted values.

In order to visually compare the superiority of our model, we compared the fitting performance of the traditional models and DrugNet on certain samples, as shown in Figure 4, Figure 5, and Figure 6, which are visualizations of the fitting performance of the K-P and Weibull models and DrugNet on samples 1, 24, 35, and 39, respectively. Samples 1, 24, 35, and 39 are those whose release curves clearly do not conform to the exponential function. The discrete points are the actual data, and the curves are the predicted values. It can be seen that due to the form and parameter limitations of the explicit mathematical formulas in traditional semi-empirical models, the shape of the fitted curve is similar. However, it is difficult to obtain good fitting results for samples with significant changes in the curve’s shape. The neural network DrugNet uses the relevant features of the PLGA carriers, drug molecules, and time points as inputs; focuses on the factors that affect the drug release curve; and implicitly learns their interaction relationships by training the network parameters. Therefore, the neural network can capture the nonlinear relationships in drug release data better. Therefore, neural networks perform better in handling nonlinear changes in the data, demonstrating greater adaptability and advantages than traditional models.

Moreover, when using traditional semi-empirical models for curve fitting, this is carried out sample by sample, and all of the data on each sample are used. However, when training the neural network, we use a random 80% portion of the data divided based on all of the sample time points to train the model and achieve better results. This indicates that DrugNet achieved better results than the semi-empirical models with less than 20% of data, demonstrating a better fitting ability and universality.

## 4. Conclusions

This study demonstrates the advantage of DrugNet in fitting the in vitro drug release curves for PLGA vectors by constructing a dataset. It can quickly learn effective features and rules from the training data, not only performing well on training data but also maintaining a good performance on unseen validation data. Compared with the semi-empirical K-P and Weibull models, the MSE of this model decreases by 20.994 and 1.561, respectively, and $R^{2}$ increases by 0.036 and 0.005, demonstrating its strong ability to fit drug release curves and capture the nonlinear relationships in drug release data better. It performs better in handling nonlinear changes in the data, has stronger adaptability and advantages than traditional models, and overcomes the limitations of the mathematical expressions in traditional semi-empirical models.

This work explores the application of neural networks to in vitro drug release curves based on the copolymer carriers. Our work investigates the relationship between PLGA’s characteristics, the drug’s characteristics, and the release curves, which provides a reference for similar tasks (predicting drug dissolution, harmful substances’ concentrations, etc.). It is worth noting that our model is currently segmented based on time points, and future research could improve the data segmentation method to make the training data more in line with the real drug release curves and enhance the model’s ability to generalize across curves further. Our goal is to extend the model to other types of copolymer carriers and different drug molecules to establish a more comprehensive in vitro drug release prediction framework.

## Figures and Tables

**Table 1:** The details of the drug release dataset.

Drug Name	Source	Release Time Scale	Point Number
Camptothecin	[14]	20–40 (h)	33
Insulin	[15]	20–40 (h)	37
Insulin	[15]	20–40 (h)	33
Lansoprazole	[16]	20–40 (h)	35
Loperamide	[17]	40–80 (h)	35
Tacrolimus	[18]	200–400 (h)	35
Rhein	[19]	10–20 (h)	35
VEGF	[20]	400–1000 (h)	35
Calcitriol	[21]	100–200 (h)	35
Cerebrolysin	[22]	10–20 (h)	35
18-β-glycyrrhetinic acid	[23]	40–80 (h)	35
18-β-glycyrrhetinic acid	[23]	40–80 (h)	35
Dopamine	[24]	100–200 (h)	35
DNaseI	[25]	200–400 (h)	35
Netilmicin	[26]	200–400 (h)	35
Cisplatin	[27]	200–400 (h)	35
Thienorphine	[28]	200–400 (h)	8
Flavopiridol	[29]	40–80 (h)	8
Gemcitabine	[30]	100–200 (h)	11
ASC-J9	[31]	200–400 (h)	21
Felodipine	[32]	100–200 (h)	11
Emtricitabine	[33]	200–400 (h)	15
Temazolamide	[34]	100–200 (h)	10
Sildenafil	[35]	10–20 (h)	8
Caryota mitis Profilin	[36]	200–400 (h)	16
Paclitaxel	[37]	100–200 (h)	6
Docetaxel	[38]	100–200 (h)	10
Ibuprofen	[39]	0–10 (h)	8
Cytarabine	[40]	20–40 (h)	7
Curcumin	[41]	100–200 (h)	13
Curcumin	[41]	100–200 (h)	12
Camptothecin	[42]	400–1000 (h)	25
Carvacrol	[43]	20–40 (h)	10
Shikonin	[44]	40–80 (h)	6
Sertaconazole	[45]	20–40 (h)	7
Evodiamine	[46]	100–200 (h)	18
Amikacin	[47]	10–20 (h)	8
Rutin	[48]	40–80 (h)	10
Salinomycin	[49]	400–1000 (h)	20

**Table 2:** The table of MSE results.

Sample ID	Source	K-P	Weibull	DrugNet
1	[14]	27.629	17.656	2.459
2	[15]	46.751	15.429	6.175
3	[15]	3.34	25.584	4.767
4	[16]	14.638	4.699	5.708
5	[17]	63.628	21.99	6.719
6	[18]	5.557	5.647	3.146
7	[19]	49.384	9.898	5.586
8	[20]	2.746	1.875	15.638
9	[21]	47.466	19.981	2.201
10	[22]	16.702	12.046	20.248
11	[23]	8.957	4.159	3.05
12	[23]	2.944	1.832	3.461
13	[24]	9.013	6.269	3.987
14	[25]	32.36	24.187	2.616
15	[26]	1.554	4.597	2.049
16	[27]	39.187	29.911	8.516
17	[28]	6.631	1.129	12.594
18	[29]	26.738	9.755	45.325
19	[30]	3.147	12.502	17.529
20	[31]	73.67	6.492	9.065
21	[32]	8.806	28.458	3.737
22	[33]	3.032	13.482	37.949
23	[34]	7.718	11.115	35.374
24	[35]	26.282	33.064	15.017
25	[36]	6.708	3.505	4.098
26	[37]	25.273	7.703	46.85
27	[38]	30.68	11.166	2.797
28	[39]	4.523	6.805	16.989
29	[40]	40.652	3.897	13.56
30	[41]	29.529	12.047	8.091
31	[41]	5.611	8.519	7.5
32	[42]	60.37	27.353	19.663
33	[43]	104.735	0.918	12.06
34	[44]	2.705	3.831	11.066
35	[45]	176.78	52.661	46.605
36	[46]	20.648	11.06	5.895
37	[47]	123.463	10.139	25.502
38	[48]	124.397	11.997	15.682
39	[49]	54.805	87.494	10.724
avg	-	34.327	14.894	**13.333**

**Table 3:** The table of $R^{2}$ results.

Sample ID	Source	K-P	Weibull	DrugNet
1	[14]	0.786	0.863	0.981
2	[15]	0.928	0.976	0.991
3	[15]	0.996	0.966	0.994
4	[16]	0.967	0.989	0.987
5	[17]	0.892	0.963	0.989
6	[18]	0.99	0.99	0.994
7	[19]	0.924	0.985	0.991
8	[20]	0.992	0.994	0.954
9	[21]	0.891	0.954	0.995
10	[22]	0.949	0.963	0.938
11	[23]	0.984	0.993	0.995
12	[23]	0.98	0.988	0.977
13	[24]	0.952	0.966	0.979
14	[25]	0.931	0.948	0.994
15	[26]	0.996	0.987	0.994
16	[27]	0.914	0.934	0.981
17	[28]	0.982	0.997	0.966
18	[29]	0.974	0.990	0.955
19	[30]	0.996	0.983	0.976
20	[31]	0.888	0.99	0.986
21	[32]	0.988	0.96	0.995
22	[33]	0.993	0.971	0.917
23	[34]	0.975	0.964	0.885
24	[35]	0.933	0.915	0.961
25	[36]	0.981	0.990	0.989
26	[37]	0.957	0.987	0.920
27	[38]	0.94	0.978	0.994
28	[39]	0.961	0.941	0.854
29	[40]	0.942	0.994	0.981
30	[41]	0.957	0.983	0.988
31	[41]	0.988	0.981	0.983
32	[42]	0.902	0.956	0.968
33	[43]	0.856	0.999	0.983
34	[44]	0.991	0.988	0.964
35	[45]	0.808	0.943	0.949
36	[46]	0.937	0.966	0.982
37	[47]	0.843	0.987	0.967
38	[48]	0.718	0.973	0.964
39	[49]	0.829	0.727	0.967
avg	-	0.934	0.965	**0.970**

**Table 4:** The standard deviations in the MSE and $R^{2}$ of different models on all samples.

Evaluation Indicators	K-P	Weibull	Ours
MSE	39.317	15.882	**12.662**
$R^{2}$	0.065	0.046	**0.031**

## Data Availability

The datasets used in this paper are publicly available.

## References

[1] Trucillo P. (2021). Drug carriers: Classification, administration, release profiles, and industrial approach. Processes.

[2] Ghitman J., Biru E.I., Stan R., Iovu H. (2020). Review of hybrid PLGA nanoparticles: Future of smart drug delivery and theranostics medicine. Mater. Des..

[3] Danhier F., Ansorena E., Silva J.M., Coco R., Le Breton A., Préat V. (2012). PLGA-based nanoparticles: An overview of biomedical applications. J. Control. Release.

[4] Mir M., Ahmed N., ur Rehman A. (2017). Recent applications of PLGA based nanostructures in drug delivery. Colloids Surf. B Biointerfaces.

[5] Lagreca E., Onesto V., Di Natale C., La Manna S., Netti P.A., Vecchione R. (2020). Recent advances in the formulation of PLGA microparticles for controlled drug delivery. Prog. Biomater..

[6] Barzegar-Jalali M. (2008). Kinetic analysis of drug release from nanoparticles. J. Pharm. Pharm. Sci..

[7] Jahromi L.P., Ghazali M., Ashrafi H., Azadi A. (2020). A comparison of models for the analysis of the kinetics of drug release from PLGA-based nanoparticles. Heliyon.

[8] Sun Y., Peng Y., Chen Y., Shukla A.J. (2003). Application of artificial neural networks in the design of controlled release drug delivery systems. Adv. Drug Deliv. Rev..

[9] Adekoya O.C., Yibowei M.E., Adekoya G.J., Sadiku E.R., Hamam Y., Ray S.S. (2022). A mini-review on the application of machine learning in polymer nanogels for drug delivery. Mater. Today Proc..

[10] Hassanzadeh P., Atyabi F., Dinarvand R. (2019). The significance of artificial intelligence in drug delivery system design. Adv. Drug Deliv. Rev..

[11] Rafienia M., Amiri M., Janmaleki M., Sadeghian A. (2010). Application of artificial neural networks in controlled drug delivery systems. Appl. Artif. Intell..

[12] Petrović J., Ibrić S., Betz G., Đurić Z. (2012). Optimization of matrix tablets controlled drug release using Elman dynamic neural networks and decision trees. Int. J. Pharm..

[13] Koshari S.H., Chang D.P., Wang N.B., Zarraga I.E., Rajagopal K., Lenhoff A.M., Wagner N.J. (2019). Data-driven development of predictive models for sustained drug release. J. Pharm. Sci..

[14] Householder K.T., DiPerna D.M., Chung E.P., Wohlleb G.M., Dhruv H.D., Berens M.E., Sirianni R.W. (2015). Intravenous delivery of camptothecin-loaded PLGA nanoparticles for the treatment of intracranial glioma. Int. J. Pharm..

[15] Malathi S., Nandhakumar P., Pandiyan V., Webster T.J., Balasubramanian S. (2015). Novel PLGA-based nanoparticles for the oral delivery of insulin. Int. J. Nanomed..

[16] Alai M., Lin W.J. (2015). Application of nanoparticles for oral delivery of acid-labile lansoprazole in the treatment of gastric ulcer: In vitro and in vivo evaluations. Int. J. Nanomed..

[17] O’Donnell A., Moollan A., Baneham S., Ozgul M., Pabari R.M., Cox D., Kirby B.P., Ramtoola Z. (2015). Intranasal and intravenous administration of octa-arginine modified poly (lactic-co-glycolic acid) nanoparticles facilitates central nervous system delivery of loperamide. J. Pharm. Pharmacol..

[18] Shin S.B., Cho H.Y., Kim D.D., Choi H.G., Lee Y.B. (2010). Preparation and evaluation of tacrolimus-loaded nanoparticles for lymphatic delivery. Eur. J. Pharm. Biopharm..

[19] Yuan Z., Gu X. (2015). Preparation, characterization, and in vivo study of rhein-loaded poly (lactic-co-glycolic acid) nanoparticles for oral delivery. Drug Des. Dev. Ther..

[20] Chereddy K.K., Lopes A., Koussoroplis S., Payen V., Moia C., Zhu H., Sonveaux P., Carmeliet P., des Rieux A., Vandermeulen G. (2015). Combined effects of PLGA and vascular endothelial growth factor promote the healing of non-diabetic and diabetic wounds. Nanomed. Nanotechnol. Biol. Med..

[21] Ramalho M.J., Loureiro J.A., Gomes B., Frasco M.F., Coelho M.A., Pereira M.C. (2015). PLGA nanoparticles as a platform for vitamin D-based cancer therapy. Beilstein J. Nanotechnol..

[22] Ruozi B., Belletti D., Sharma H.S., Sharma A., Muresanu D.F., Mössler H., Forni F., Vandelli M.A., Tosi G. (2015). PLGA nanoparticles loaded cerebrolysin: Studies on their preparation and investigation of the effect of storage and serum stability with reference to traumatic brain injury. Mol. Neurobiol..

[23] Darvishi B., Manoochehri S., Kamalinia G., Samadi N., Amini M., Mostafavi S.H., Maghazei S., Atyabi F., Dinarvand R. (2015). Preparation and antibacterial activity evaluation of 18-*β*-glycyrrhetinic acid loaded PLGA nanoparticles. Iran. J. Pharm. Res. IJPR.

[24] Pahuja R., Seth K., Shukla A., Shukla R.K., Bhatnagar P., Chauhan L.K.S., Saxena P.N., Arun J., Chaudhari B.P., Patel D.K. (2015). Trans-blood brain barrier delivery of dopamine-loaded nanoparticles reverses functional deficits in parkinsonian rats. ACS Nano.

[25] Osman R., Kan P.L., Awad G., Mortada N., Abd-Elhameed E.S., Alpar O. (2011). Enhanced properties of discrete pulmonary deoxyribonuclease I (DNaseI) loaded PLGA nanoparticles during encapsulation and activity determination. Int. J. Pharm..

[26] Kolate A., Kore G., Lesimple P., Baradia D., Patil S., Hanrahan J.W., Misra A. (2015). Polymer assisted entrapment of netilmicin in PLGA nanoparticles for sustained antibacterial activity. J. Microencapsul..

[27] Menale C., Piccolo M.T., Favicchia I., Aruta M.G., Baldi A., Nicolucci C., Barba V., Mita D.G., Crispi S., Diano N. (2015). Efficacy of piroxicam plus cisplatin-loaded PLGA nanoparticles in inducing apoptosis in mesothelioma cells. Pharm. Res..

[28] Yang Y., Xie X.Y., Mei X.G. (2016). Preparation and in vitro evaluation of thienorphine-loaded PLGA nanoparticles. Drug Deliv..

[29] Ren H., Han M., Zhou J., Zheng Z.F., Lu P., Wang J.J., Wang J.Q., Mao Q.J., Gao J.Q., Ouyang H.W. (2014). Repair of spinal cord injury by inhibition of astrocyte growth and inflammatory factor synthesis through local delivery of flavopiridol in PLGA nanoparticles. Biomaterials.

[30] Joshi G., Kumar A., Sawant K. (2014). Enhanced bioavailability and intestinal uptake of Gemcitabine HCl loaded PLGA nanoparticles after oral delivery. Eur. J. Pharm. Sci..

[31] Verderio P., Pandolfi L., Mazzucchelli S., Marinozzi M.R., Vanna R., Gramatica F., Corsi F., Colombo M., Morasso C., Prosperi D. (2014). Antiproliferative effect of ASC-J9 delivered by PLGA nanoparticles against estrogen-dependent breast cancer cells. Mol. Pharm..

[32] Shah U., Joshi G., Sawant K. (2014). Improvement in antihypertensive and antianginal effects of felodipine by enhanced absorption from PLGA nanoparticles optimized by factorial design. Mater. Sci. Eng. C.

[33] Singh G., Pai R.S. (2014). Optimization (Central Composite Design) and Validation of HPLC Method for Investigation of Emtricitabine Loaded Poly (lactic-co-glycolic acid) Nanoparticles: In Vitro Drug Release and In Vivo Pharmacokinetic Studies. Sci. World J..

[34] Jain D.S., Athawale R.B., Bajaj A.N., Shrikhande S.S., Goel P.N., Nikam Y., Gude R.P. (2014). Unraveling the cytotoxic potential of Temozolomide loaded into PLGA nanoparticles. DARU J. Pharm. Sci..

[35] Ghasemian E., Vatanara A., Rouholamini Najafabadi A., Rouini M.R., Gilani K., Darabi M. (2013). Preparation, characterization and optimization of sildenafil citrate loaded PLGA nanoparticles by statistical factorial design. DARU J. Pharm. Sci..

[36] Xiao X., Zeng X., Zhang X., Ma L., Liu X., Yu H., Mei L., Liu Z. (2013). Effects of Caryota mitis profilin-loaded PLGA nanoparticles in a murine model of allergic asthma. Int. J. Nanomed..

[37] Li M., Czyszczon E.A., Reineke J.J. (2013). Delineating intracellular pharmacokinetics of paclitaxel delivered by PLGA nanoparticles. Drug Deliv. Transl. Res..

[38] Shi W., Zhang Z.j., Yuan Y., Xing E.m., Qin Y., Peng Z.j., Zhang Z.p., Yang K.y. (2013). Optimization of parameters for preparation of docetaxel-loaded PLGA nanoparticles by nanoprecipitation method. J. Huazhong Univ. Sci. Technol. [Med. Sci.].

[39] Bonelli P., Tuccillo F.M., Federico A., Napolitano M., Borrelli A., Melisi D., Rimoli M.G., Palaia R., Arra C., Carinci F. (2012). Ibuprofen delivered by poly (lactic-co-glycolic acid)(PLGA) nanoparticles to human gastric cancer cells exerts antiproliferative activity at very low concentrations. Int. J. Nanomed..

[40] Yadav K.S., Sawant K.K. (2010). Modified nanoprecipitation method for preparation of cytarabine-loaded PLGA nanoparticles. Aaps Pharmscitech..

[41] Nair K.L., Thulasidasan A.K.T., Deepa G., Anto R.J., Kumar G.V. (2012). Purely aqueous PLGA nanoparticulate formulations of curcumin exhibit enhanced anticancer activity with dependence on the combination of the carrier. Int. J. Pharm..

[42] Blum J.S., Weller C.E., Booth C.J., Babar I.A., Liang X., Slack F.J., Saltzman W.M. (2011). Prevention of K-Ras-and Pten-mediated intravaginal tumors by treatment with camptothecin-loaded PLGA nanoparticles. Drug Deliv. Transl. Res..

[43] Iannitelli A., Grande R., Di Stefano A., Di Giulio M., Sozio P., Bessa L.J., Laserra S., Paolini C., Protasi F., Cellini L. (2011). Potential antibacterial activity of carvacrol-loaded poly (DL-lactide-co-glycolide)(PLGA) nanoparticles against microbial biofilm. Int. J. Mol. Sci..

[44] Fu J., You L., Sun D., Zhang L., Zhao J., Li P. (2024). Shikonin-loaded PLGA nanoparticles: A promising strategy for Psoriasis Treatment. Heliyon.

[45] Eleraky N.E., Attia M.A., Safwat M.A. (2024). Sertaconazole-PLGA nanoparticles for management of ocular keratitis. J. Drug Deliv. Sci. Technol..

[46] Zou L., Chen F., Bao J., Wang S., Wang L., Chen M., He C., Wang Y. (2016). Preparation, characterization, and anticancer efficacy of evodiamine-loaded PLGA nanoparticles. Drug Deliv..

[47] Sabaeifard P., Abdi-Ali A., Soudi M.R., Gamazo C., Irache J.M. (2016). Amikacin loaded PLGA nanoparticles against Pseudomonas aeruginosa. Eur. J. Pharm. Sci..

[48] Gagliardi A., Paolino D., Costa N., Fresta M., Cosco D. (2021). Zein-vs PLGA-based nanoparticles containing rutin: A comparative investigation. Mater. Sci. Eng. C.

[49] Irmak G., Öztürk M.G., Gümüşderelioğlu M. (2020). Salinomycin encapsulated PLGA nanoparticles eliminate osteosarcoma cells via inducing/inhibiting multiple signaling pathways: Comparison with free salinomycin. J. Drug Deliv. Sci. Technol..

